# Two gonad-infecting species of *Philometra* (Nematoda: Philometridae) from marine fishes off the northern coast of Australia

**DOI:** 10.1051/parasite/2015008

**Published:** 2015-02-06

**Authors:** František Moravec, Diane P. Barton

**Affiliations:** 1 Institute of Parasitology, Biology Centre of the Academy of Sciences of the Czech Republic Branišovská 31 370 05 České Budějovice Czech Republic; 2 Fisheries Research, Department of Primary Industries & Fisheries, Berrimah Farm Darwin Northern Territory 0801 Australia Aquatic Ecology & Management, Research Institute for the Environment and Livelihoods, Charles Darwin University Darwin Northern Territory 0801 Australia

**Keywords:** Philometridae, New species, Australia

## Abstract

Two different gonad-infecting species of *Philometra* Costa, 1845 were collected from the ovary of marine perciform fishes, the blackspotted croaker *Protonibea diacanthus* (Sciaenidae) and the John’s snapper *Lutjanus johnii* (Lutjanidae), from off the northern coast of Australia. Nematodes (males and females) from *P*. *diacanthus* represent a new taxon, *Philometra protonibeae* n. sp., which is mainly characterized by the body length of the males (3.37–3. 90 mm), broad, equally long spicules (length 126–141 μm) and the shape and structure of the gubernaculum with a dorsally lamellate distal tip. The nematodes (only females) from *L*. *johnii* may represent an undescribed species, but, because of the absence of conspecific males, they could not be specifically identified. *Philometra protonibeae* is the fifth nominal gonad-infecting species of this genus recorded from marine fishes in Australian waters and the seventh species of these parasites described from fishes of the family Sciaenidae.

## Abstract (fr)

Deux espèces différentes de *Philometra* Costa, 1845, parasites des gonades de poissons, ont été collectées des ovaires de poissons perciformes marins, *Protonibea diacanthus* (Sciaenidae) et *Lutjanus johnii* (Lutjanidae), au large de la côte nord de l’Australie. Les nématodes (mâles et femelles) de *P. diacanthus* représentent un nouveau taxon, *Philometra protonibeae* n. sp., qui se caractérise principalement par la longueur du corps des mâles (3.37-3.90 mm), des spicules larges et de même longueur (126-141 μm) et la forme et la structure du gubernaculum, qui a une pointe distale dorsalement lamellaire. Les nématodes (seulement femelles) de *L. johnii* peuvent représenter une espèce non décrite, mais, en raison de l’absence des mâles conspécifiques, n’ont pas pu être identifiés au niveau de l’espèce. *Philometra protonibeae* est la cinquième espèce nominale de ce genre infectant les gonades qui est mentionnée de poissons marins dans les eaux australiennes et la septième espèce décrite de poissons de la famille Sciaenidae.

## Introduction

The knowledge of the fauna of philometrid nematodes (Philometridae) parasitizing fishes in Australian waters remains fragmentary. To date, a total of 16 valid nominal species of these parasites belonging to the genera *Buckleyella* Rasheed, 1963 (1 species), *Philometra* Costa, 1845 (11 species), *Philometroides* Yamaguti, 1935 (3 species) and *Spirophilometra* Parukhin, 1971 (1 species) have been recorded in this region [3, 14–16, 28, 32]. An additional 13 nominal species of *Philometra* parasitizing marine fishes were reported from off New Caledonia, South Pacific [12, 18–21], which may also occur in Australian waters. Beveridge et al. [1] have recently reported a 45% overall similarity between the fauna of trypanorhynch metacestodes in teleosts of the Great Barrier Reef and New Caledonia and a similar situation may occur in the case of philometrids.

During recent helminthological investigations of some marine fishes off the northern Australian coast, philometrid nematodes were collected from the ovary of two species of perciform fishes, the blackspotted croaker *Protonibea diacanthus* (Lacépède) (Sciaenidae) and the John’s snapper *Lutjanus johnii* (Bloch) (Lutjanidae). A close examination revealed that they represent two different species of *Philometra*, one from the former host, which represents a new species, and the other from the latter host which cannot be identified to species due to the absence of the males. Both of these forms are described herein. The two aforementioned hosts are tropical marine fishes with an Indo-West Pacific distribution and are targeted by commercial and recreational fishermen [2].

## Materials and methods

Fish were collected by hook and line from six locations along the northern Australia coastline (Table 1). All fish were anaesthetized, killed and stored in ice for transportation back to the laboratory for further examination. At the laboratory, each fish was measured (total length; mm), the body cavity opened and all internal organs removed. Internal organs were placed in a sealed plastic bag and frozen until examined for parasites. At this time, the ovaries were separated from the remaining internal organs, placed in a Petri dish of physiological saline and opened under a dissecting microscope and examined for parasites.

**Table 1.** Collection localities, no. of female fish, average total length and number of infected fish examined.

Fish Species	Location caught (coordinates)	No. of female fish examined	Average total length (mm) (range)	No. of fish infected
*Protonibea diacanthus*	Camden Sound, WA	16	646.4	2
	(16° 11′31.20ʺ S; 124° 32′31.20ʺ E)		(524–920)	
	Fenton Patches, NT	4	1030	1
	(12° 11′30.00ʺ S; 130° 42′30.00ʺ E)		(930–1150)	
	Ruby Island, NT	4	772.5	1
	(12°6′16.86ʺ S; 131° 21′6.05ʺ E)		(430–1210)	
*Lutjanus johnii*	Bynoe Harbour, NT	6	320.8	2
	(12° 37′26.31ʺ S; 130° 30′1.87ʺ E)		(260–380)	
	Lee Point, NT	2	345	1
	(12° 19′51.03ʺ S; 130° 53′1.43ʺ E)		(330–360)	
	Nicol Island, NT	3	696.7	2
	(13° 27′8.27ʺ S; 136° 15′10.64ʺ E)		(640–770)	

The nematodes obtained were washed in physiological saline and were then fixed and preserved in 70% ethanol. For light microscopical examination, the nematodes were cleared with glycerine. Drawings were made with the aid of a Zeiss drawing attachment. Specimens used for scanning electron microscopy (SEM) were postfixed in 1% osmium tetroxide (in phosphate buffer), dehydrated through a graded acetone series, critical-point-dried and sputter-coated with gold; they were examined using a JEOL JSM-7401F scanning electron microscope at an accelerating voltage of 4 kV (GB low mode). All measurements are in micrometres unless otherwise indicated. The fish nomenclature adopted follows FishBase [2].

## *Philometra protonibeae* n. sp. (Figs. 1–3)

urn:lsid:zoobank.org:act:D1FACC35-7178-4FC2-BC97-44FC5B06C9E8

**Figure 1.**
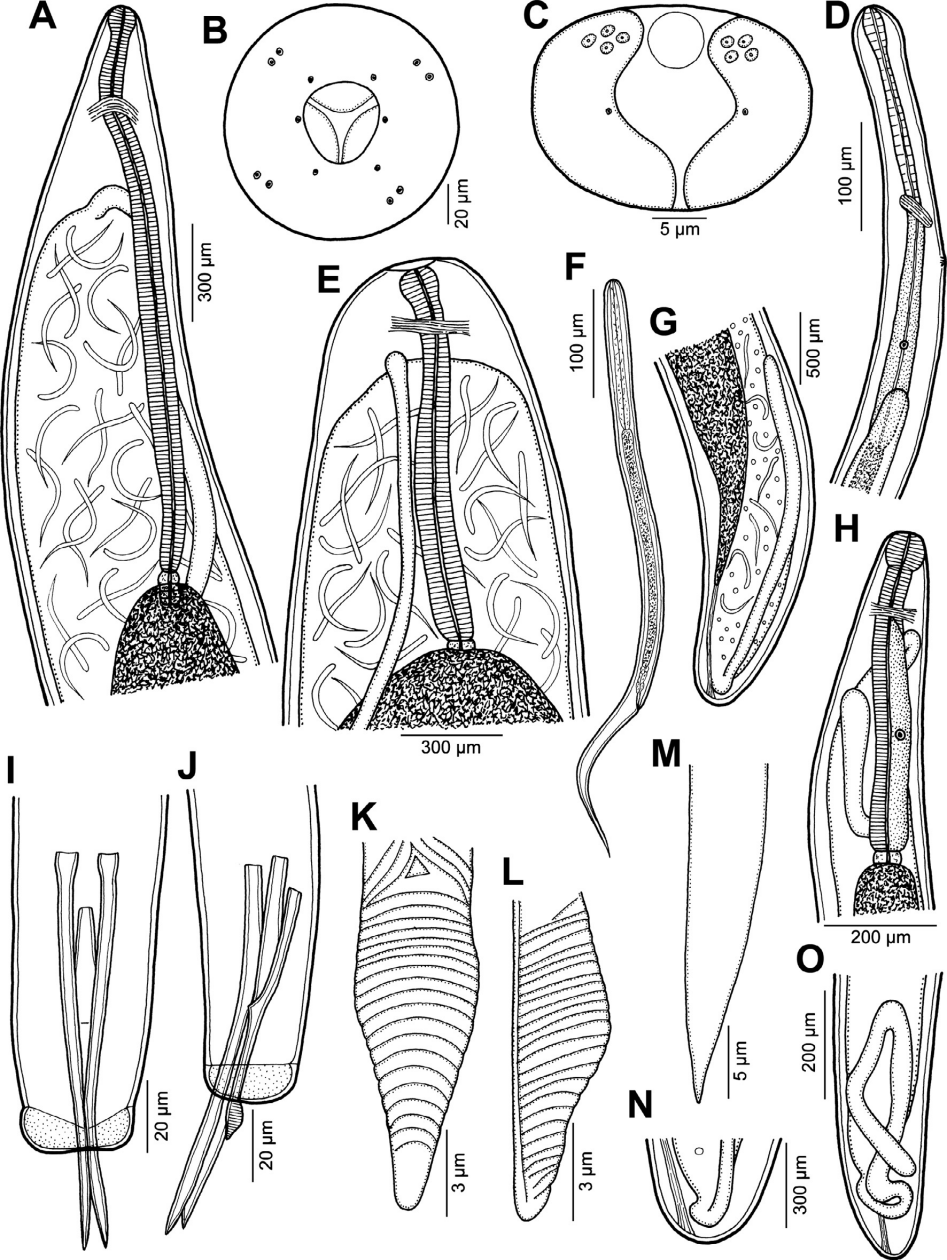
*Philometra protonibeae* n. sp. from *Protonibea diacanthus*. A: Anterior end of gravid female, lateral view. B: Cephalic end of gravid female, apical view. C: Caudal end of male, apical view. D: Anterior end of male, lateral view. E: Anterior end of gravid female (another specimen), lateral view. F: Larva from uterus, lateral view. G: Posterior end of gravid female, lateral view. H: Anterior end of nongravid female, lateral view. I, J: Posterior end of male, ventral and lateral views. K, L: Distal end of gubernaculum, dorsal and lateral views. M: Distal end of spicule, lateral view. N: Caudal end of gravid female, lateral view. O: Posterior end of nongravid female, lateral view.

Type host: Blackspotted croaker, *Protonibea diacanthus* (Lacépède) (Sciaenidae, Perciformes) (body length 1150 mm).

Site of infection: Ovary.

Type locality: Fenton Patches, 35 km North-West of Darwin Harbour, Northern Territory (NT), Australia (collected 4 July 2013).

Other localities: Camden Sound, approximately 120 km North of Derby, Western Australia (WA); Ruby Island, near Cape Hotham, approximately 100 km North-East of Darwin Harbour, NT, Australia.

Prevalence: 25%.

Type specimens: Holotype, allotype and paratypes in the South Australian Museum, Adelaide, Australia (Cat. Nos. AHC 47547–47551); other paratypes in the Helminthological Collection of the Institute of Parasitology, Biology Centre of the Academy of Sciences of the Czech Republic, České Budějovice (Cat. No. N-1077).

Etymology: The specific name of this nematode relates to the genitive form of the generic name of the host.

### Description

**Figure 2.**
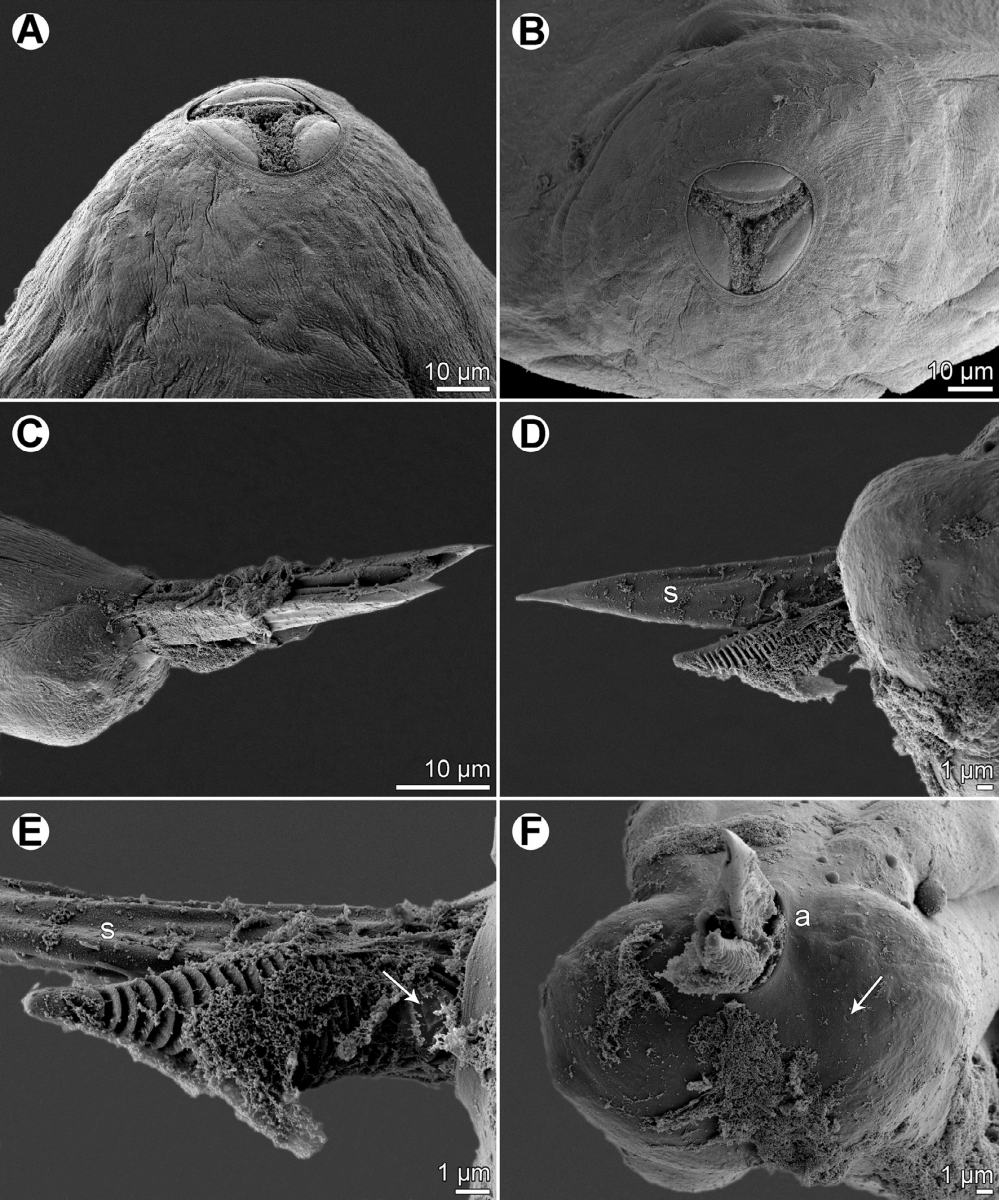
*Philometra protonibeae* n. sp. from *Protonibea diacanthus*, scanning electron micrographs. A, B: cephalic end of subgravid female, subapical and apical views. C: Caudal end of male with protruded spicules and gubernaculum, lateral view. D: Protruded distal ends of spicule and gubernaculum, lateral view. E: Distal end of gubernaculum, dorsal view (arrow indicates elevated triangular structure). F: Caudal end of male, apical view (arrow indicates phasmid). *Abbreviations*: a, group of four flat caudal papillae; s, spicule.

**Figure 3.**
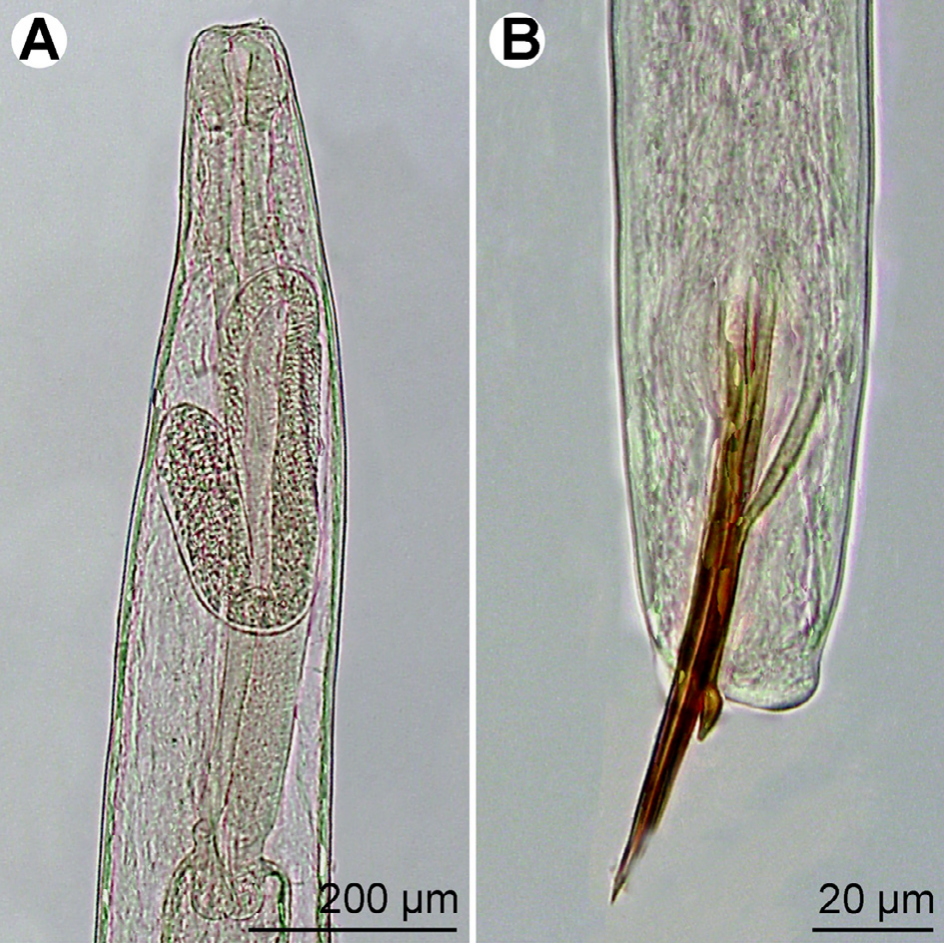
*Philometra protonibeae* n. sp. from *Protonibea diacanthus*, light microscope micrographs. A: Anterior end of nongravid female, lateral view. B: Posterior end of male, lateral view.

*Male* (eight specimens; measurements of holotype in parentheses): body whitish, filiform, tapering to both ends, 3.37–3.90 (3.67) mm long, maximum width at middle 69–78 (75); anterior part of body with slight constriction just posterior to cephalic end (Fig. 1D); body width at this constriction 39–42 (42). Maximum width/body length 1:49–54 (1:49); width of cephalic end 42–48 (48), that of posterior end 27–36 (30). Cuticle smooth. Cephalic end rounded. Oral aperture small, oval, surrounded by 14 cephalic papillae arranged in two circles: external circle formed by four submedian pairs of papillae; internal circle formed by four submedian and two lateral papillae. Small lateral amphids just posterior to lateral papillae of internal circle. Oesophagus 375–555 (375) long, maximum width 21–24 (21), slightly inflated at anterior end; posterior end of muscular oesophagus overlapped by well-developed oesophageal gland with large cell nucleus in middle (Fig. 1D); anterior oesophageal inflation 39 (39) long and 21 (21) wide. Nerve ring, excretory pore and oesophageal nucleus 186–213 (213), 210–264 (264) and 381–384 (381), respectively, from anterior extremity. Testis reaching anteriorly to about mid-way between oesophageal nucleus and posterior end of oesophagus (Fig. 1D). Posterior end of body blunt, with two broad, reniform caudal mounds well separated dorsally (Figs. 1C, 2F). Four pairs of very flat, hardly visible caudal papillae close to each other situated on either side of cloacal aperture on mound (Figs. 1C, 2F). Pair of small phasmids present at about middle of each mound (Figs. 1C, 2F). Spicules equally long, with somewhat expanded proximal and sharply pointed distal tips; distal region of spicules markedly (*c*. 4 μm) wide in lateral view (Figs. 1I, J, M, 2C–F); length of spicules 126–141 (138), comprising 3.4–3.8% (3.8%) of body length. Gubernaculum narrow, 78–90 (87) long, with slight dorsal curvature of anterior region; length of anterior curved part 36–48 (42), representing 43–53% (48%) of entire gubernaculum length; posterior end of gubernaculum with distinct dorsal protuberance bearing numerous transverse lamella-like structures (Figs. 1I–L, 2C–F). Length ratio of gubernaculum and spicules 1:1.40–1.62 (1:1.59). Spicules and gubernaculum brownish, anterior region of gubernaculum colourless (Fig. 3).

*Gravid female* (numerous body fragments of six specimens; measurements of allotype in parentheses): body fragments dark-brown. Body length not determined; largest fragment, of specimen with missing posterior end, 427 mm long; maximum body width 0.93–2.18 (1.16) mm; cephalic end 122–544 (136) wide. Cuticle smooth. Cephalic papillae small, indistinct when viewed laterally (Fig. 1A, E). Oral aperture oval, surrounded by outer circle of four pairs of small submedian cephalic papillae and inner circle of six single papillae (two lateral and four submedian) (Fig. 1B). Amphids indistinct. Bottom of mouth formed by lobes of three oesophageal sectors (Figs. 1B, 2A, B). Oesophagus including anterior bulbous inflation 1.56–2.49 (2.29) mm long; anterior inflation 120–177 (136) long and 93–177 (109) wide; maximum width of posterior, markedly narrow part of oesophagus 95–163. Oesophageal gland indistinct (Fig. 1A, E). Nerve ring 299–408 (381) from anterior extremity; oesophageal nucleus not found. Small ventriculus 54–69 (57) long and 69–105 wide. Posterior end of body rounded, 163–422 wide, without any caudal projections (Fig. 1G, N). Length of intestinal ligament 490–544. Ovaries reflexed, situated near body extremities (Fig. 1A, E, G). Uterus filled with numerous larvae and eggs. Larvae (*n* = 5) 533–606 long, maximum width 15–18; length of oesophagus 144–174 (26–31% of body length), of tail 159–189 (30–33%) (Fig. 1F).

*Subgravid female* (body fragments of four specimens): body length not determined; length of longest body fragment 45 mm; maximum width of fragments 503–966. Width of cephalic end 109–190. Oesophagus, including anterior bulbous inflation, 1.09–1.95 mm long, maximum width 75–82; anterior inflation 85–122 long and 82–95 wide. Nerve ring 204–313 from anterior extremity; oesophageal nucleus not observed. Ventriculus 30–36 long and 72–90 wide. Intestinal ligament 272–408 long. Uterus filled with eggs. Posterior end of body rounded, 163–190 wide, without any caudal projections.

*Nongravid female* (one mature specimen): body length 25 mm, maximum width 231; width of anterior end 95. Entire oesophagus 639 long, 68 wide. Anterior oesophageal inflation 68 long, 68 wide. Oesophageal gland distinct, extending between nerve ring and end of oesophagus (Fig. 1H); nerve ring and oesophageal nucleus 150 and 326 from anterior extremity, respectively. Vulva and vagina absent. Uterus empty. Caudal end rounded (Fig. 1O).

### Remarks

To date, the following six valid nominal gonad-infecting species of *Philometra* have been reported from fishes of the family Sciaenidae: *P*. *carolinensis* Moravec, de Buron & Roumillat, 2006 from *Cynoscion nebulosus* (Cuvier) and *Menticirrhus americanus* (Linnaeus), and *P*. *floridensis* Moravec, Fajer-Ávila & Bakenhaster, 2010 from *Sciaenops ocellatus* (Linnaeus), both off the Atlantic coast of the USA [11–13, 17]; *P*. *johnii* Moravec & Ali, 2013 from *Johnius dussumieri* (Cuvier) and *Johnius* sp. in the Persian Gulf and off northern Australia, respectively [8, 14]; *P*. *otolithi* Moravec & Manoharan, 2013 from *Otolithes ruber* (Bloch & Schneider) in the Bay of Bengal and the Persian Gulf [9, 22–24]; and *P*. *sciaenae* Yamaguti, 1941 from *Pennahia argentata* (Houttuyn) off Japan [27, 31, 34]. Moreover, *P*. *lateolabracis* (Yamaguti, 1935), a specific parasite of the Lateolabracidae, was reported from fishes belonging to six genera of the Sciaenidae (including *Protonibea diacanthus*) [7, 30], but, in view of the paper by Quiazon et al. [30], the nematodes from these hosts were evidently misidentified. An additional, poorly described species *P*. *rajani* Mukherjee, 1963, a parasite of *Eleutheronema tetradactylum* (Shaw) (Polynemidae), was reported from the ovary of some sciaenid fishes off India [6, 29] but later designated as a *species inquirenda* [24].

All of the above-mentioned valid species are easily distinguishable from *P*. *protonibeae* n. sp. by the morphology of the males and, in particular, the structure of the distal end of the gubernaculum. In contrast to the new species, the gubernaculum of *P*. *carolinensis* and *P*. *floridensis* is smooth, not transversely lamellate, but possesses a distinct dorsal barb on its distal end. The gubernaculum of *P*. *johnii*, *P*. *otolithi* and *P*. *sciaenae* is provided with many marked transverse lamella-like structures forming a distinct dorsal protuberance visible in lateral view, as in the new species. However, the dorsal protuberance on the gubernaculum of *P*. *johnii* and *P*. *otolithi* appears to be single in lateral view but in fact consists of two dorsolateral parts separated from each other by a smooth longitudinal field when observed dorsally. Quiazon et al. [31] illustrated a bipartite dorsal protuberance on the gubernaculum of *P*. *sciaenae*, where no median smooth field between the two parts of the protuberance was present, but the lamella-like structures on the protuberance were interrupted dorsally by a median longitudinal line. In contrast to *P*. *johnii*, *P*. *otolithi* and *P*. *sciaenae*, the protuberance on the gubernaculum of *P*. *protonibeae* is simple, without a division into two parts, and its lamella-like structures are not interrupted dorsally.

As mentioned above, *P*. *lateolabracis* has previously been reported from the gonads of *Protonibea diacanthus* off India [4]. Since only specifically unidentifiable females were available, it may well be that they were conspecific with *P*. *protonibeae* n. sp. Although the male morphology of *P*. *lateolabracis*, as redescribed by Quiazon et al. [30] based on specimens from the type host, is similar to that of *P*. *protonibeae* and these two species resemble each other in the lengths of spicules and gubernaculum (65–130 and 50–93 μm in *P*. *lateolabracis* vs. 126–141 and 78–90 μm in *P*. *protonibeae*), the males of the new species are somewhat longer (3.37–3.90 mm vs. 2.07–2.73 mm). However, the two species differ distinctly in the structure of the gubernaculum: in *P*. *protonibeae* the narrowed distal tip posterior to the dorsal protuberance is markedly shorter and transversely lamellate (vs. elongate, approximately as long as the protuberance and without transverse lamellae); the dorsally uninterrupted transverse lamellae extend anteriorly only to the proximal end of the dorsal protuberance (vs. extending anteriorly into the region anterior to the protuberance); and a few (two or three) oblique lamellae and a small median triangular structure are present at the dorsal region anterior to the proximal end of the protuberance (Figs. 1K, 2E) (no such structures were observed in *P*. *lateolabracis*, but the gubernaculum of this species has not yet been studied in dorsal view). The above-mentioned triangular dorsal structure on the gubernaculum has not been described for any other species of *Philometra*. *Philometra protonibeae* and *P*. *lateolabracis* also differ in the shape of the distal regions of the spicules, which are markedly broad (*c*. 4 μm) in lateral view in the former species, but relatively narrow (*c*. 2 μm wide) in lateral view in the latter species.

Females of *Philometra lateolabracis* have recently been reported by Sethi et al. [33] from the caudal fins of two sciaenid fishes, *Otolithes ruber* (Bloch et Schneider) and *Pennahia macrophthalmus* (Bleeker) (= *P*. *anea* (Bloch)), in the Bay of Bengal, off India. However, this is an evident misidentification. As can be seen in the published photographs of the worms from *O*. *ruber*, the structure of the oesophagus (presence of a conspicuously large anterior oesophageal bulb) and the presence of marked cuticular ornamentations on the body indicate that these nematodes, at least those from *O*. *ruber*, represent the philometrid *Clavinemoides annulatus* Moravec, Khosheghbal & Pazooki, 2013, a species recently described from the caudal fin of the same host species (*O*. *ruber*) in the Persian Gulf off Iran [22].

## *Philometra* sp. (Fig. 4)

Host: John’s snapper, *Lutjanus johnii* (Bloch) (Lutjanidae, Perciformes) (average total length 498 mm; range 260–770 mm).

**Figure 4.**
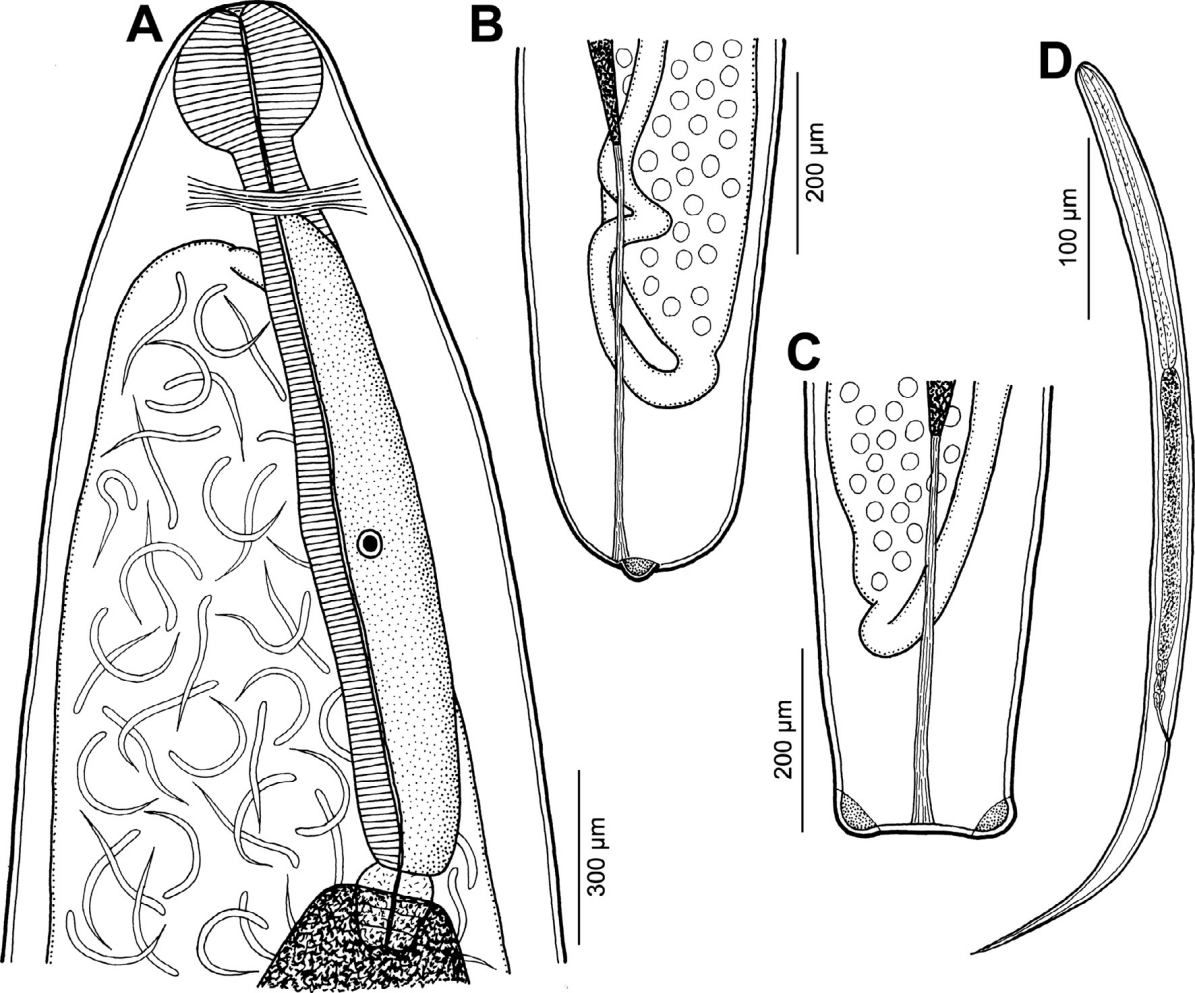
*Philometra* sp. from *Lutjanus johnii*. A: Anterior end of largest gravid female, lateral view. B, C: Posterior end of smaller gravid female, lateral and dorsoventral views. D: Larva from uterus, lateral view.

Site of infection: Ovary.

Localities: Bynoe Harbour, approximately 100 km South-West of Darwin Harbour; Lee Point, on the north-eastern side of outer Darwin Harbour; and Nicol Island, near Groote Eylandt, Gulf of Carpentaria, all off NT, Australia (collected between 2 December 2013 and 10 March 2014).

Prevalence: 45.5%.

Specimens: Deposited in the South Australian Museum, Adelaide, Australia (Cat. Nos. AHC 47552–47553).

### Description

*Gravid female* (body fragments of one complete and one incomplete larvigerous specimens): body yellowish, 47.00 mm long, maximum width 748; body of incomplete specimen (caudal end missing) 139 mm long, maximum width 1.77 mm; cephalic end 204–258 wide. Cuticle smooth. Cephalic papillae small, indistinct when viewed laterally (Fig. 4A). Oesophagus, including anterior bulbous inflation, 1.56–1.65 mm long; anterior inflation distinct, 95–231 long and 122–272 wide; maximum width of posterior part of oesophagus, including gland, 190–218. Oesophageal gland well developed, opens into oesophagus just posterior to nerve ring, with large cell nucleus in middle (Fig. 4A). Nerve ring and oesophageal nucleus 218–367 and 1197–1088 from anterior extremity, respectively. Ventriculus small, 30–41 long, 54–163 wide. Posterior end of body rounded, 245 wide, with 2 large, lateral papilla-like caudal projections (Fig. 4B, C). Length of intestinal ligament 639. Ovaries reflexed, situated near body extremities (Fig. 4A–C). Uterus filled with numerous larvae and eggs. Larvae (*n* = 5) 531–758 long, maximum width 15–18; length of oesophagus 156–168 (29–31% of body length), of tail 162–180 (31–32%) (Fig. 4D).

*Subgravid female* (one ovigerous specimen): body 32.00 mm long, maximum width 680. Width of cephalic end 204. Length of oesophagus could not be established; anterior bulbous inflation 231 long, 218 wide. Posterior end of body rounded, 204 wide, with 2 papilla-like caudal projections. Uterus contains several eggs.

*Nongravid female* (two mature specimens; measurement of smaller specimen in parentheses): body length 11.97 (2.49) mm, maximum width 245 (33); width of anterior end 136 (109). Entire oesophagus 1.24 (–) long and 95 (–) wide. Anterior oesophageal inflation 54 (30) long, 60 (18) wide. Nerve ring and oesophageal nucleus 218 (–) and 938 (–) from anterior extremity, respectively. Vulva and vagina absent in larger specimen, but present in smaller specimen, situated 1.70 mm from anterior extremity (at 68% of body length). Uterus empty. Caudal end rounded, without any caudal projections.

### Remarks

To date, the following eight gonad-infecting species of *Philometra* have been described from different *Lutjanus* spp.: *P*. *latispicula* Moravec, Bakenhaster & Fajer-Ávila, 2014, *P*. *longispicula* Moravec, Bakenhaster & Fajer-Ávila, 2014 and *P*. *synagridis* Moravec, Bakenhaster & Fajer-Ávila, 2014 in the Gulf of Mexico (USA) [10]; *P*. *argentimaculati* Moravec & Manoharan, 2014 and *P*. *fulvi* Moravec & Manoharan, 2014 in the Bay of Bengal (off India) [25]; *P*. *brevicollis* Moravec & Justine, 2012 and *P*. *mira* Moravec & Justine, 2012 in New Caledonian waters [20]; and *P*. *carponotati* Moravec et Diggles, 2014 from off northern Australia [15].

All of the above-mentioned species are distinguishable from each other mainly on the basis of male morphology (in fact, *P*. *mira* and *P*. *synagridis* are known only by their males), whereas the morphology of conspecific gravid and subgravid females is rather uniform, not enabling reliable species identification. The presence of fairly large, papilla-like caudal projections and a well-developed anterior oesophageal bulb in gravid females of the present material from *L*. *johnii* indicates that these nematodes may belong to an undescribed species (caudal projections are missing or much smaller in other species parasitizing *Lutjanus* spp. and their oesophageal bulb is usually less developed). However, in view of the absence of males and the high degree of host specificity in gonad-infecting species of *Philometra* when different species are found in congeneric fish hosts in the same locality [10, 25, 26], we refrain from establishing a new species for these nematodes until conspecific males are available.

Kardousha [5] reported philometrid females identified as *Philometra lateolabracis* from the ovary of the same host species, *L*. *johnii*, in the Persian Gulf, but in view of the revisionary paper of Quiazon et al. [30] on *P*. *lateolabracis*, this would appear to be a misidentification.
